# Matrine Restores Porcine-Origin β-Lactam-Resistant *Escherichia coli* to Cefepime and Cefquinome: Association with Impaired Biofilm Formation and β-Lactamase Production

**DOI:** 10.3390/antibiotics15050494

**Published:** 2026-05-14

**Authors:** Bo Yang, Wen Yang, Bingyan Hu, Jingchao Zhao, Hui Deng, Lingxian Yi, Penghua Jian, Zelin Hong, Daojin Yu

**Affiliations:** Fujian Key Laboratory of Traditional Chinese Veterinary Medicine and Animal Health, College of Animal Sciences, Fujian Agriculture and Forestry University, Fuzhou 350002, China; ybvet@fafu.edu.cn (B.Y.); gumiyoung@163.com (W.Y.); 19959653783@163.com (B.H.); 18950411126@163.com (J.Z.); 000q816016@fafu.edu.cn (H.D.); lingxian_yi@outlook.com (L.Y.); 832342889@139.com (P.J.); fafuvet200204@outlook.com (Z.H.)

**Keywords:** Matrine, biofilm, β-lactamases, cefepime, cefquinome, *Escherichia coli*, antibiotic resistance reversal, transcriptomics

## Abstract

**Background:** The in vivo efficacy and mechanisms of matrine (MT) in reversing β-lactam resistance in *E. coli* remain unclear. **Methods**: β-lactam-resistant *E. coli* strains were treated with MT both in vitro and in a murine intestinal colonization model. Phenotypic changes (MIC, morphology, growth, biofilm, β-lactamase) were evaluated, and transcriptomic profiles were analyzed. **Results**: MT at sub-inhibitory concentrations significantly and concentration-dependently reduced the MICs of β-lactam-resistant *E. coli* strains by 2- to 32-fold in vitro. This reduction was also confirmed in vivo, and its magnitude became more pronounced as the number of doses increased. MT treatment dispersed bacterial aggregates and dissipated extracellular matrix, but did not alter the morphology of individual bacteria. At concentrations above 1024 μg/mL, MT significantly inhibited bacterial growth; lower concentrations (≤512 μg/mL) had no effect. Notably, MT inhibited biofilm formation and β-lactamase production both in vitro and in vivo. **Conclusions:** MT restored the susceptibility of β-lactam-resistant *E. coli* to cefepime and cefquinome. This effect was associated with suppression of biofilm formation and β-lactamase production, which correlated with the downregulation of key genes (*ycgR*, *pgaB*, *pgaD*, *bla_TEM_* and *bla_CTX-M_*).

## 1. Introduction

*Escherichia coli* (*E. coli*), an important opportunistic pathogen, poses significant threats to both public and animal health. Pathogenic strains of *E. coli* are capable of causing a diverse array of infections and pathological changes, such as diarrhea, hemorrhagic colitis, edema, urethritis, airsacculitis, arthritis and septicemia [1]. In livestock and poultry, these infections manifest as increased mortality rates, reduced production efficiency, and elevated veterinary costs, imposing considerable economic burdens on the farming industry. β-lactam antibiotics, with their potent antibacterial activity and excellent safety record, remain the first-line agents against colibacillosis in veterinary practice. Unfortunately, the overuse and misuse of β-lactams have resulted in widespread resistance to these drugs in *E. coli* [2], a problem that severely undermines the clinical efficacy of this antibiotic class. Given the high cost and prolonged development cycles for novel β-lactam antibiotics, reinvigorating the efficacy of existing β-lactams represents a highly promising and pragmatic anti-infective strategy.

For centuries, traditional Chinese medicine has been employed to treat infectious diseases. Among these medicinal herbs, *Sophora flavescens* Aiton (nomenclature verified via World Flora Online, March 2026) is indicated for the treatment of heat dysentery, reddish and white vaginal discharge, vulvar swelling and itching, damp sore, scabies, tinea and leprosy according to the *Chinese Pharmacopoeia* [3]. Additionally, *Sophora flavescens* is recommended for the treatment of damp-heat diarrhea and dysentery, scabies and tinea in livestock and poultry, as specified in the *Chinese Veterinary Pharmacopoeia* [4]. The screening of such medicinal herbs for resistance-reversal agents, combined with existing antibiotics, represents a promising approach to combating antimicrobial resistance [5]. Matrine (MT, CAS 519-02-8) is a quinolizidine alkaloid extracted from *Sophora flavescens* and *Sophora alopecuroides* L. (nomenclature verified via World Flora Online, March 2026). It is also a major bioactive constituent of the total alkaloids of *Sophora alopecuroides* [6]. Previous studies have suggested that MT has the potential to restore the susceptibility of resistant *E. coli* to certain antibiotics, including ampicillin, streptomycin, gentamicin, and tetracycline [7,8,9,10]. Similar resistance-reversing effects have been reported for the total alkaloids of *Sophora alopecuroides*, which re-sensitize resistant *E. coli* to cefotaxime, ceftazidime, ampicillin and amoxicillin [6,11,12,13]. The proposed mechanisms are multifaceted and putatively involve: (i) inhibiting the protective function of the QnrS protein via binding to its Loop B; (ii) downregulating the transcriptional levels of key resistance genes (ARGs), such as *bla_TEM_*, *bla_SHV_, tetA*, *tetB*, *tetC*; (iii) suppressing efflux pump activity, particularly the AcrAB-TolC system; and (iv) interfering with the quorum sensing (QS) system. Given that MT is the major bioactive constituent of these total alkaloids, it is reasonable to infer that it may exert a similar resistance-reversing effect against β-lactam resistance in *E. coli*, a hypothesis yet to be experimentally verified. Even so, while the resistance-reversing effects of MT and the total alkaloids of *Sophora alopecuroides* against β-lactam-resistant *E. coli* have been observed in vitro, their in vivo efficacy remains unconfirmed. Moreover, the molecular mechanisms underlying MT-mediated reversal of β-lactam resistance in *E. coli* have not yet been fully elucidated.

The present study aimed to systematically evaluate the potential of MT to reverse β-lactam resistance in porcine-derived *E. coli* both in vitro and in vivo. The key resistance-associated phenotypes were assessed, including minimum inhibitory concentration (MIC), bacterial morphology and growth kinetics, biofilm formation, and β-lactamase production. Transcriptomic profiling was further employed to elucidate the molecular mechanisms underlying MT-mediated reversal of β-lactam resistance. Our findings are expected to provide new mechanistic insights into this MT-mediated resistance reversal and support MT as a promising β-lactam resistance-reversal agent.

## 2. Results

### 2.1. β-Lactam Resistance Profiles of Clinical E. coli Strains and Their Intestinal Colonization Ability

Among the 193 clinical *E. coli* isolates, the prevalence of resistance to the seven β-lactam antibiotics was as follows: amoxicillin (98.45%), cefazolin (46.11%), cefaclor (42.49%), cefotaxime (41.45%), ceftiofur (40.41%), cefepime (18.13%), and cefquinome (16.06%). Twenty-four strains were resistant to all seven β-lactams and are listed in Table 1. For these strains, the MIC of MT was 8192 μg/mL. The biofilm formation ability of 24 β-lactam-resistant strains was shown in Table 2. Strains C10, C12, C20, C25, C26, and C29 showed OD_600_ values greater than two-fold higher than the control, indicating strong biofilm formation. Table 3 showed the efflux pump phenotype of 24 β-lactam-resistant strains. For strains C3, C14, C20, C26, and C29, the mean ratio of MIC__Control_ to MIC__CCCP treatment_ was ≥4, indicating their efflux pump-positive phenotype. As shown in Table 4, β-lactamase was detected in all strains, with levels ranging from 51.24 to 96.31 pg/mL. The presence of five antibiotic ARGs in 24 β-lactam-resistant strains was shown in Table 5. Strains C15, C26, and C29 were found to carry all 5 ARGs. Based on the combined data from Table 1, Table 2, Table 3, Table 4 and Table 5, strains C26 and C29 displayed the most diverse β-lactam resistance profiles among the 24 β-lactam-resistant strains.

**Figure 1:**
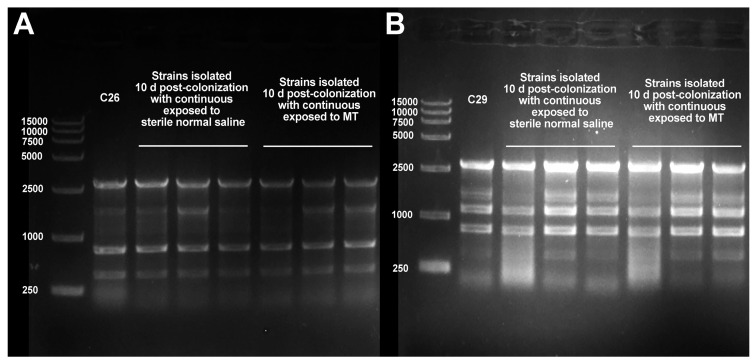
ERIC-PCR identification of colonized *E. coli* recovered from mouse feces on day 10 post-inoculation. Profiles confirm stable intestinal colonization of strain C26 (**A**) and strain C29 (**B**), validating their suitability for subsequent in vivo evaluation of MT-mediated resistance reversal.

As shown in Figure 1, the Enterobacterial Repetitive Intergenic Consensus Polymerase Chain Reaction (ERIC-PCR) profiles of *E. coli* from murine fecal samples on day 10 after colonization matched those of strains C26 and C29. This indicates successful colonization by both strains for 10 consecutive days, despite their continuous exposure to either sterile saline or MT. All mice remained asymptomatic after 3 consecutive days of gavage.

Based on their comprehensive resistance profiles and confirmed ability to colonize the murine intestine, strains C26 and C29 were selected for further evaluation of the resistance-reversing effects of MT on β-lactam-resistant *E. coli*.

### 2.2. Resistance-Reversing Effects of MT on β-Lactam-Resistant E. coli

The changes in MICs of seven tested β-lactams against the 24 β-lactam-resistant *E. coli* strains after MT treatment were shown in Table 6. Upon exposure to MT at 4096 μg/mL for 48 h, the susceptibility to cefepime and cefquinome was restored in most resistant strains (cefepime: 22/24, cefquinome: 21/24), with MIC values decreasing by 2- to 128-fold; partial restoration of susceptibility to cefazolin, cefaclor, cefotaxime, and ceftiofur was observed in subsets of resistant strains (cefazolin: 4/24; cefaclor: 7/24; cefotaxime: 6/24; ceftiofur: 6/24), accompanied by a 2- to 16-fold reduction in MICs. Conversely, MT did not reverse resistance to amoxicillin in any of the resistant strains.

**Figure 2:**
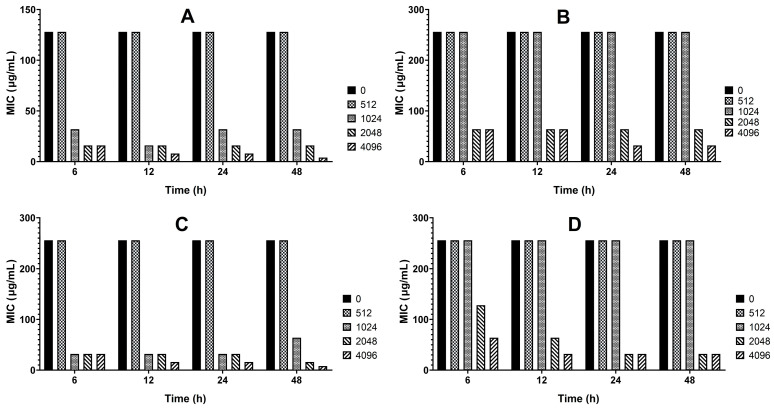
Changes in MICs of cefepime and cefquinome against strains C26 and C29 after MT treatment in vitro, determined by broth microdilution method. (**A**) strain C26 treated with cefepime; (**B**) strain C26 treated with cefquinome; (**C**) strain C29 treated with cefepime; (**D**) strain C29 treated with cefquinome.

**Figure 3:**
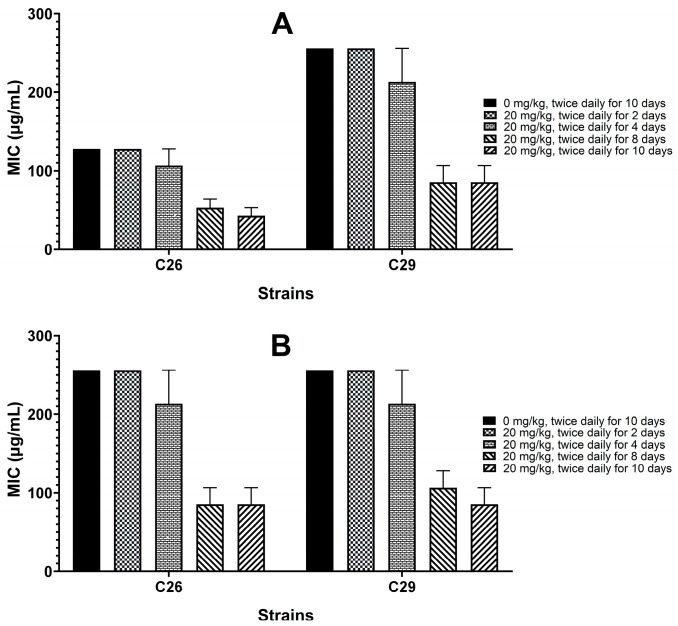
Changes in MICs of cefepime and cefquinome against strains C26 and C29 after MT treatment in vivo, determined by broth microdilution method. (**A**) cefepime; (**B**) cefquinome.

As shown in Figure 2, treatment with sub-inhibitory concentrations of MT reduced the MICs of cefepime and cefquinome against strains C26 and C29 by 2- to 32-fold in vitro. This reduction exhibited concentration- and time-dependent patterns. Notably, the MT-induced reduction in MICs was also pronounced in vivo, where its magnitude increased with the number of doses administered (Figure 3).

**Figure 4:**
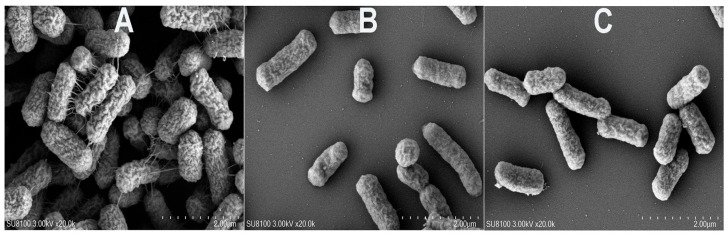
The morphological changes in strain C26 observed by SEM after MT treatment, where panels (**A**–**C**) represent the morphology of MT-untreated strain C26, MT-treated strain C26 (512 μg/mL), and MT-treated strain C26 (2048 μg/mL), respectively.

The morphological changes in strain C26 after MT treatment were shown in Figure 4. Compared with the untreated control, the MT-treated strain exhibited a significant decrease in bacteria count per microscopic field, accompanied by dispersed bacteria and dissipation of the unidentified extracellular matrix between cells. However, the morphology of individual bacteria remained unchanged. Additionally, comparison between the low (512 μg/mL) and high (2048 μg/mL) MT concentration groups revealed no significant differences in bacterial count or cellular morphology per field of view.

**Figure 5:**
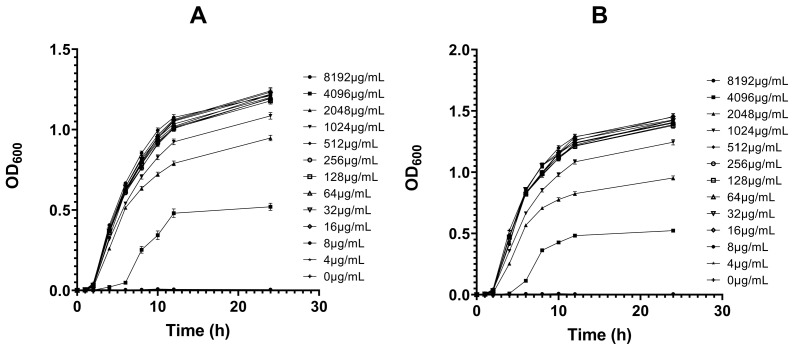
Growth kinetics of MT-treated (various concentrations) and untreated strains C26 and C29 monitored by optical density (OD600) over time, where panels (**A**,**B**) correspond to strains C26 and C29, respectively.

As shown in Figure 5, the growth kinetics of MT-treated and untreated strains C26 and C29 showed similar temporal patterns during the logarithmic (2–6 h) and stationary (12 h and thereafter) phases. MT at 1024 μg/mL or higher significantly inhibited the growth of both strains, whereas concentrations ≤ 512 μg/mL had no effect. The inhibitory effect on the growth of strains C26 and C29 exhibited a concentration-dependent pattern.

**Figure 6:**
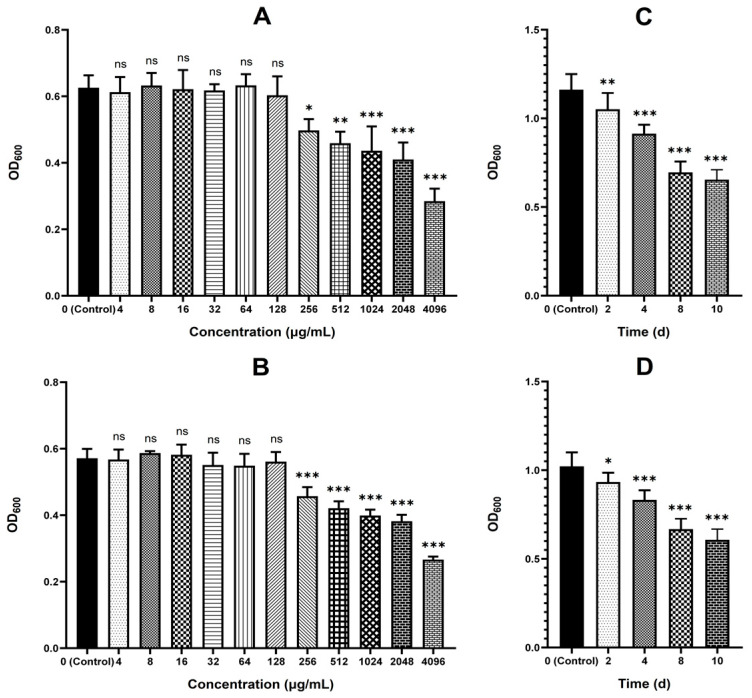
Changes in the biofilm formation ability of strains C26 and C29 after MT treatment, quantified by crystal violet assay. Panels (**A**–**D**) correspond to the changes in the biofilm formation ability of strain C26 (in vitro), strain C29 (in vitro), strain C26 (in vivo), and strain C29 (in vivo), respectively. ns, no significant difference; * *p* < 0.05, ** *p* < 0.01, *** *p* < 0.001 versus the untreated control group.

The changes in the biofilm formation ability of strains C26 and C29 after MT treatment were shown in Figure 6. Compared to the untreated control (0 μg/mL MT), treatment with MT at concentrations ≥ 256 μg/mL resulted in significant, concentration-dependent decreases in the absorbance (OD_600_) of crystal violet-stained biofilms (*p* < 0.05) for both strains. In vivo studies showed that oral administration of MT to mice at 20 mg/kg twice daily for 2, 4, 8, and 10 days also significantly decreased the OD_600_ of crystal violet-stained biofilms relative to the untreated group (*p* < 0.05).

**Figure 7:**
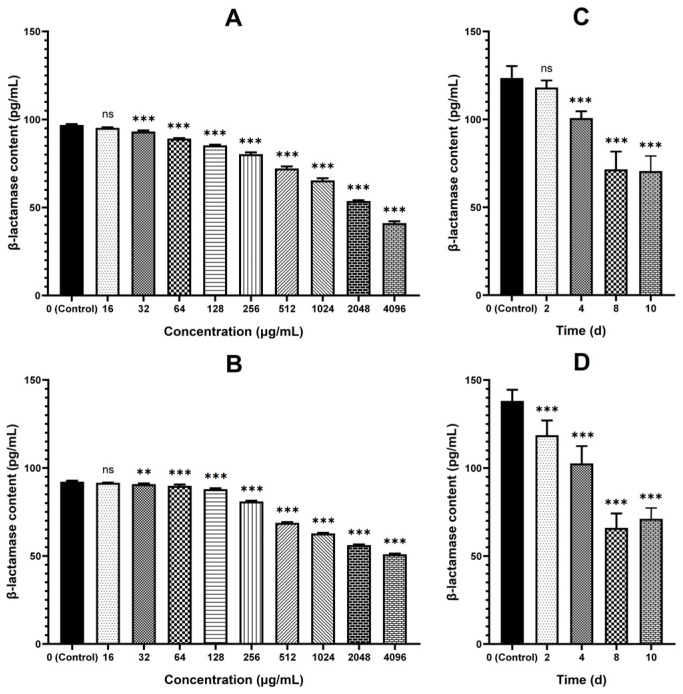
Changes in the β-lactamase production of strains C26 and C29 after MT treatment, quantified by ELISA. Panels (**A**–**D**) correspond to the changes in the β-lactamase production of strain C26 (in vitro), strain C29 (in vitro), strain C26 (in vivo), and strain C29 (in vivo), respectively. ns, no significant difference; ** *p* < 0.01, *** *p* < 0.001 versus the untreated control group.

As shown in Figure 7, the β-lactamase production of strains C26 and C29 decreased after MT treatment. In vitro, MT at concentrations of 32 μg/mL and above significantly decreased the β-lactamase production in a dose-dependent manner (*p* < 0.01), whereas concentrations of 16 μg/mL and below showed no effect. Similarly, the β-lactamase production of both intestinal-colonized strains was also significantly reduced (*p* < 0.05) in mice that received oral MT (20 mg/kg, twice daily) for 4, 8, and 10 consecutive days.

### 2.3. Differentially Expressed Genes and Their Functional Annotations in β-Lactam-Resistant E. coli Strain After MT Treatment

**Figure 8:**
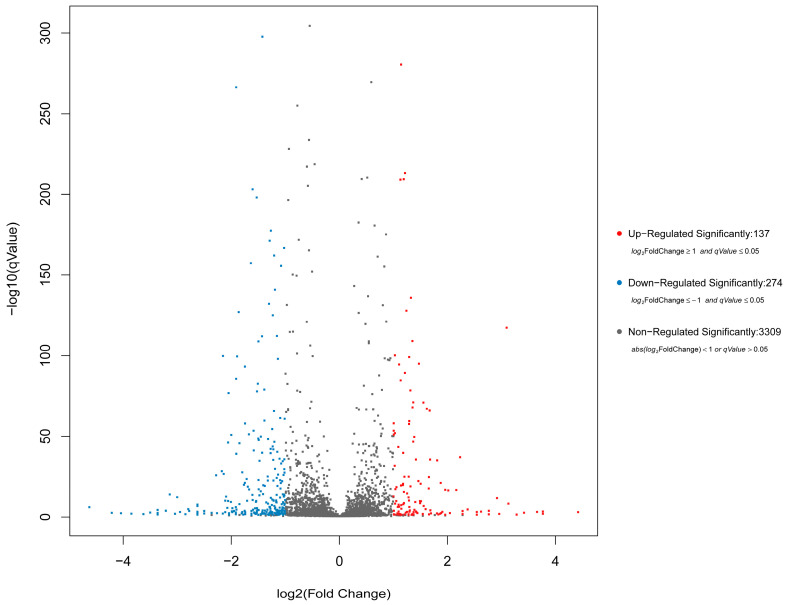
Changes Volcano plots showing DEGs with adjusted *p* < 0.05 and |log_2_ fold change| ≥ 1, identified by RNA sequencing.

**Figure 9:**
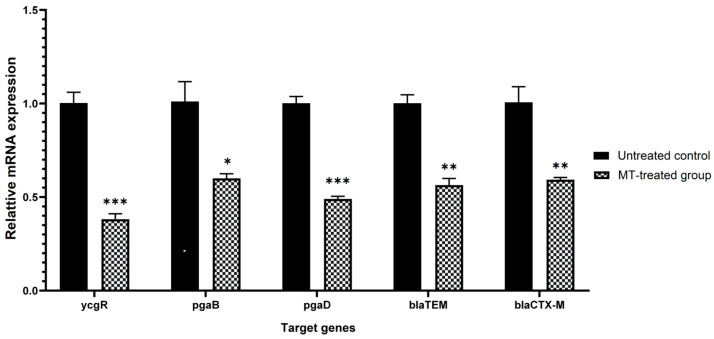
Effects of MT on the relative expression of three biofilm-related genes (*ycgR*, *pgaB*, and *pgaD*) and two β-lactamase-encoding genes (*bla_TEM_* and *bla_CTX-M_*) detected by qRT-PCR (*, *p* < 0.05; **, *p* < 0.01; *** *p* < 0.001). Data are presented as mean ± SD from three technical replicates.

Differentially Expressed Genes (DEGs) were highlighted in the volcano plots with adjusted *p* < 0.05 and |log_2_FC| ≥ 1 (Figure 8). A total of 137 genes were significantly up-regulated and 274 genes were down-regulated in the MT-treated group compared with the untreated control. Several DEGs potentially involved in biofilm formation were significantly downregulated. These included *ycgR* (encodes flagellar brake protein), *pgaB* (encodes poly-beta-1,6-N-acetyl-D-glucosamine N-deacetylase [EC:3.5.1.-]), and *pgaD* (encodes biofilm PGA synthesis protein PgaD). The quantitative real-time polymerase chain reaction (qRT-PCR) results showed that the expression levels of *ycgR*, *pgaB*, and *pgaD* in strain C26 were significantly downregulated (*p* < 0.05) after MT treatment (Figure 9), which was consistent with the transcriptome data. Regarding the regulation of β-lactamases, a downregulation trend was observed for the key genes (*bla_TEM_* and *bla_CTX-M_*) after MT treatment, although it was not statistically significant compared to the untreated control. Interestingly, the expression level of the two genes was unequivocally confirmed as significant (*p* < 0.01) by the more sensitive qRT-PCR assay (Figure 9).

**Figure 10:**
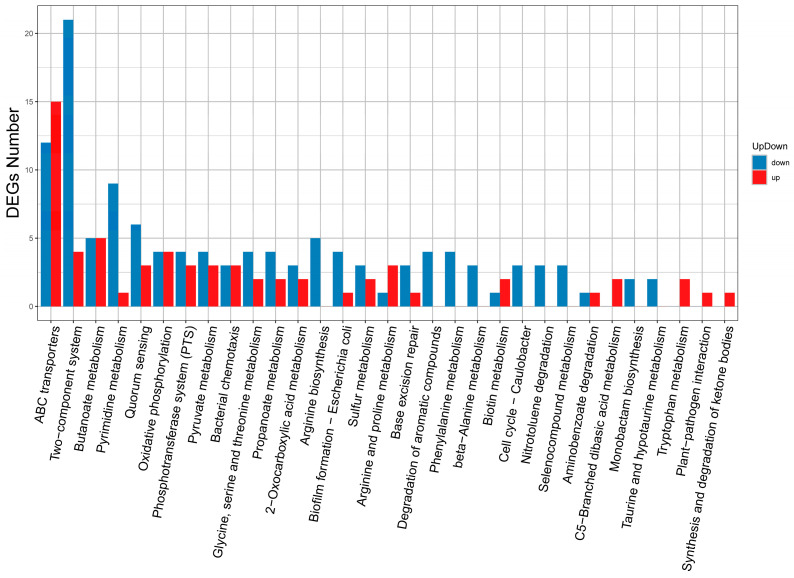
KEGG pathway enrichment analysis of DEGs in the MT-treated group compared with the untreated control, based on RNA sequencing data.

**Figure 11:**
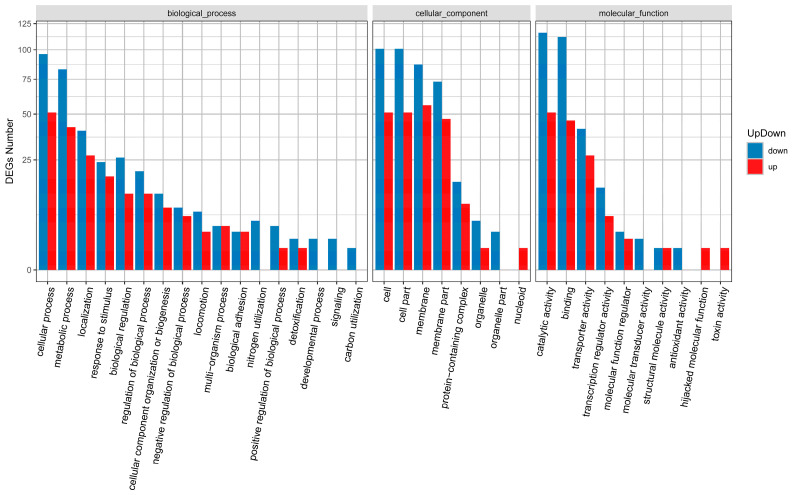
Functional enrichment analysis of DEGs in the MT-treated group compared with the untreated control, based on RNA sequencing data.

Kyoto Encyclopedia of Genes and Genomes (KEGG) pathway enrichment analysis is presented in Figure 10. Compared to the untreated control, the MT-treated group exhibited significant enrichment of DEGs in pathways including ATP-binding cassette transporters, two-component systems, pyrimidine metabolism, oxidative phosphorylation, QS, and biofilm formation in *E. coli*. As shown in Figure 11, Gene Ontology (GO) enrichment analysis revealed that the DEGs were primarily associated with the following categories: cellular process and metabolic process (Biological Process); cellular component and membrane (Cellular Component); as well as catalytic activity and adhesion (Molecular Function).

## 3. Discussion

β-lactam antibiotics are a major class of antibacterial agents for the treatment of *E. coli* infections. However, the resistance of pathogenic *E. coli* to β-lactams has emerged as a critical threat to public health and veterinary clinical practice. In this study, MT was shown to restore the susceptibility of β-lactam-resistant *E. coli* to cefepime and cefquinome, which is associated with impaired biofilm formation and β-lactamase production. Transcriptomic analysis further revealed that MT treatment resulted in significant downregulation of three biofilm-related genes (*ycgR*, *pgaB*, *pgaD*) and reduced expression of two β-lactamase-encoding genes (*bla_TEM_* and *bla_CTX-M_*). Significant downregulation of these key genes was also confirmed by qRT-PCR. Our findings not only support MT as a promising β-lactam resistance-reversal agent but also provide new mechanistic insights into MT-mediated β-lactam resistance reversal in *E. coli*.

β-lactam-resistant *E. coli* were screened from 193 clinical isolates based on six predefined criteria. This strategy ensured diverse β-lactam resistance profiles, allowing a comprehensive assessment of MT’s reversal effects and minimizing the risk of misinterpreting its efficacy due to limited resistance mechanisms in selected strains. *E. coli* can develop resistance to β-lactam antibiotics through multiple mechanisms, including producing β-lactamases (e.g., TEM, CTX-M) [14,15], expressing efflux pumps (e.g., AcrAB-TolC efflux pump) [14], forming biofilms [16], and mutating target proteins (penicillin-binding proteins, PBPs) [17]. Therefore, strains that harbored as many resistance profiles as possible and exhibited moderate to high-level resistance to all tested β-lactams were selected for subsequent experiments. Additionally, successful intestinal colonization in mice is another critical criterion. Intestinally colonized strains enable the subsequent evaluation of MT’s in vivo resistance-reversing effects using a model that recapitulates the host’s physiological environment. This model incorporates key factors influencing MT’s resistance-reversing effects, including interactions between *E. coli*, the intestinal microbiota, and host physiological barriers. Nevertheless, it is important to acknowledge that this screening strategy has two inherent limitations. Firstly, the requirement to meet all criteria simultaneously has severely restricted the number of qualifying strains. A mere two eligible resistant strains (C26 and C29) were obtained through screening of 193 clinical isolates. Secondly, harboring multiple resistance mechanisms in a single *E. coli* strain may render MT’s resistance-reversing effect suboptimal. When the two strains were treated with 512 μg/mL MT, their biofilm formation ability and β-lactamase production decreased significantly (Figure 6 and Figure 7); however, their MICs against cefepime and cefquinome showed no change compared with the untreated control (Figure 2).

Here, we report a systematic evaluation of MT-mediated reversal of β-lactam resistance in *E. coli*. Twenty-four β-lactam-resistant strains exhibited heterogeneous restoration of susceptibility to seven tested β-lactam antibiotics after in vitro treatment with MT. Specifically, these strains showed the most pronounced restoration of susceptibility to cefepime and cefquinome. Furthermore, partial recovery of susceptibility was observed to cefazolin, cefaclor, cefotaxime, and ceftiofur. However, no restoration of susceptibility was detected to amoxicillin at all. These findings indicate that MT may be a potential resistance-reversal agent to enhance the efficacy of cefepime and cefquinome against colibacillosis caused by β-lactam-resistant *E. coli*. To further validate these findings, we systematically evaluated the corresponding resistance-reversing effects in vitro and in vivo using representative strains C26 and C29. The result revealed that MT treatment significantly reduced the MICs against cefepime and cefquinome, as well as biofilm formation, and β-lactamase production, in both resistant strains (Figure 2, Figure 3, Figure 6 and Figure 7). Importantly, this resistance-reversing effect was further validated in the murine intestinal tract, thereby overcoming the common limitation that many potential reversal agents are only evaluated in vitro. It should be noted that while stable intestinal colonization is a prerequisite for evaluating the in vivo efficacy of MT (Figure 1), the degree of colonization did not correlate with the magnitude of β-lactam resensitization observed in vivo. This suggests that the resistance-reversing effect of MT is primarily mediated through specific pharmacological actions (e.g., inhibition of biofilm formation or suppression of β-lactamase production), rather than through indirect modulation of bacterial fitness or colonization dynamics.

Biofilm formation contributes significantly to β-lactam resistance in *E. coli* [16]. Strains C26 and C29 demonstrated strong biofilm formation ability (Table 2). MT treatment significantly reduced the biofilm biomass of both strains, as measured by crystal violet staining, in vitro and in vivo (Figure 6). The SEM results showed that MT-treated strain C26, compared with the untreated control, exhibited significantly fewer bacteria per microscopic field, appeared more dispersed, and lost the unidentified extracellular matrix on its surface. However, the morphology of individual bacteria remained unchanged. This suggests that MT treatment may facilitate the transition of this strain from the biofilm state to the planktonic state, thereby rendering it more susceptible to β-lactams. These findings, coupled with our previous results from crystal violet staining and confocal laser scanning microscopy [7], demonstrated the effective inhibitory effect of MT on the biofilm formation of *E. coli.* Previous studies have shown that both the cAMP/CRP signaling pathway and the BarA/UvrY/CsrA system are core components in the regulatory network governing bacterial biofilm formation [18,19]. Our transcriptomic data revealed that MT treatment significantly downregulated *ycgR* (a gene involved in the cAMP/CRP pathway) as well as *pgaB* and *pgaD* (components of the BarA/UvrY/CsrA system) in strain C26. YcgR is a cyclic diguanylate (c-di-GMP) effector that regulates the transition from planktonic to biofilm states [20,21,22]. Downregulation of *ycgR* likely reduces biofilm formation by relieving c-di-GMP-mediated inhibition of motility. The pgaABCD operon encodes enzymes essential for poly-N-acetyl-glucosamine (PGA) biosynthesis, a key component of the biofilm matrix [23,24]. Downregulation of *pgaB* and *pgaD* likely impairs PGA production and biofilm formation.

The production of β-lactamases is one of the important mechanisms underlying the resistance of *E. coli* to β-lactam antibiotics [14,15]. In this study, MT treatment significantly reduced β-lactamase production in strains C26 and C29 both in vitro and in vivo (Figure 7), a phenotype that coincided with the significant downregulation of the *bla_TEM_* and *bla_CTX-M_* genes as measured by qRT-PCR (Figure 9). These results provide direct evidence that MT inhibits β-lactamase production in these resistant strains, thus restoring their susceptibility to cefepime and cefquinome. Although transcriptomic analysis did not show statistically significant differential expression of *bla_TEM_* and *bla_CTX-M_*, the observed phenotypic reduction in β-lactamase production suggests possible post-transcriptional or context-dependent regulatory mechanisms. Mechanistically, these observations highlight the necessity of investigating post-transcriptional and post-translational regulatory pathways during MT-mediated resistance reversal, extending our knowledge beyond the canonical transcriptional control of ARGs.

It is noteworthy that while SEM analysis revealed no marked differences in bacterial count or cellular morphology between the 512 and 2048 μg/mL MT groups (Figure 4), the growth curve assay demonstrated a clear concentration-dependent inhibitory effect (Figure 5). This apparent discrepancy can be attributed to the distinct nature of the two assays. SEM provides a static snapshot of bacterial morphology and observable cell quantity at a single time point, whereas growth curves reflect dynamic proliferation and physiological activity over prolonged incubation. These findings suggest that MT exerts a bacteriostatic effect primarily by inhibiting bacterial growth and division rather than inducing rapid cell lysis or structural collapse. Consequently, the inhibitory effect on bacterial proliferation becomes gradually evident during continuous culture, while the differences in cell number and microstructure among different MT concentrations remain indistinguishable under short-term SEM morphological observation.

Several limitations of the present study should be noted. Although MT was found to restore the susceptibility of resistant *E*. *coli* to cefepime and cefquinome, accompanied by inhibited biofilm formation and reduced β-lactamase production, the specific molecular mechanism underlying these effects remains elusive. Specifically, the direct target genes and associated regulatory pathways of MT have not yet been identified. Additionally, the effects of MT on efflux pump overexpression (e.g., AcrAB-TolC) and PBP mutations were not evaluated, although these mechanisms also contribute to β-lactam resistance in *E. coli*. In future work, targeted experiments will be performed to identify the direct target genes and regulatory pathways of MT, and to clarify the predominant mechanism responsible for its resistance-reversing effect. Follow-up investigations will include knockout and rescue assays for genes associated with biofilm formation and β-lactamase production, qPCR analysis of efflux pump gene expression, and sequencing of PBP-encoding genes.

## 4. Materials and Methods

### 4.1. Chemicals, Bacterial Strains and Animals

MT, amoxicillin, cefazolin, cefaclor, cefotaxime, ceftiofur, cefepime, cefquinome, and streptomycin (all with purity > 98.0%) were purchased from Yuanye Bio-Technology Co., Ltd. (Shanghai, China). Carbonyl cyanide 3-chlorophenylhydrazone (CCCP, purity > 95.0%) was purchased from Macklin Inc. (Shanghai, China). Crystal violet (purity > 95.0%), glacial acetic acid (analytical grade) and methanol (analytical grade) were purchased from Sinopharm Chemical Reagent (Shanghai, China). Luria–Bertani (LB) broth, MacConkey agar, and Mueller-Hinton (MH) broth were purchased from Solarbio Science & Technology Co., Ltd. (Beijing, China). Phosphate-buffered saline (PBS) solutions (pH 7.2 and 6.0) were purchased from Coolaber Science & Technology Co., Ltd. (Beijing, China). Tris-Acetate-EDTA (TAE) buffer were purchased from Yeasen Biotechnology Co., Ltd. (Fuzhou, China).

A total of 193 clinical *E. coli* strains were isolated from anal swabs of pigs sourced from two commercial pig farms in Fujian Province, China. All strains were identified as *E. coli* by 16S rDNA sequencing analysis. *E. coli* ATCC 25922 was used as the quality control strain and was purchased from the National Institute of Pharmaceutical and Biological Products Control, Beijing, China.

Forty-two specific pathogen-free C57BL/6 mice, weighing 17–25 g, were purchased from Nordons Biotechnology Co., Ltd. (Fuzhou, China). They were housed individually in independent ventilation cages (Suzhou Fengshi Laboratory Animal Equipment Co., Ltd., Suzhou, China) under controlled environmental conditions, with temperature of 21.2 ± 3.1 °C, humidity at 60.2–76.1%, and a 12-h light/dark cycle. Antibiotic-free feed and water were provided for ad libitum. The animal experiment was approved by the Research Ethics Committee of the College of Animal Sciences, Fujian Agriculture and Forestry University (No. PZCASFAFU21031).

### 4.2. Screening of β-Lactam-Resistant E. coli Strains

The screening of β-lactam-resistant *E. coli* strains from 193 clinical isolates was performed using a multi-step strategy centered on the broth microdilution method, supplemented by phenotypic and genotypic assays including biofilm formation, efflux pump activity, β-lactamase production, presence of ARGs, and intestinal colonization.

To systematically evaluate the β-lactam resistance-reversing effects of MT, the selected strains should possess diverse β-lactam resistance profiles. Specifically, the required resistance characteristics include: (i) resistance to the tested β-lactams (preferably all tested β-lactams simultaneously) with moderate or high-level resistance (MIC values exceeding twice the resistance breakpoint); (ii) the ability to produce β-lactamase; (iii) a strong biofilm formation ability; (iv) the expression of efflux pumps (e.g., the AcrAB-TolC efflux pump); (v) the presence of ARGs encoding β-lactamase and efflux pump components. In addition, (vi) the selected strains should be capable of colonizing the murine intestinal tract, thus facilitating the evaluation of MT’s in vivo efficacy. Several experiments were performed to screen for strains that met all six of these criteria.

The susceptibility of 193 clinical isolates to β-lactams and MT was determined using the microdilution method in accordance with the Clinical and Laboratory Standards Institute (CLSI) guidelines [25]. Seven β-lactams were tested, including amoxicillin, cefazolin, cefaclor, cefotaxime, ceftiofur, cefepime, and cefquinome. The initial concentrations were 2048 μg/mL for seven the β-lactams and 32,768 μg/mL for MT. *E. coli* ATCC 25922 was used for quality control. Three biological replicates were performed for each strain. β-lactam-resistant *E. coli* strains were identified based on the determined MICs and the breakpoint values established by CLSI [25,26]. The strains with simultaneous resistance to seven β-lactams were subjected to further screening based on their biofilm formation ability, efflux pump phenotype, β-lactamase production, and carriage of ARGs.

The biofilm formation ability was evaluated via a crystal violet microtiter plate assay as follows. Briefly, a 20 μL of exponentially growing bacterial cultures was added to each well of a 96-well plate containing 180 μL of LB broth. A control well containing only 200 μL of LB broth was included. The 96-well plate was incubated at 37 °C for 24 h, washed three times with PBS (pH 7.2), and fixed with methanol for 15 min. Then, 100 μL of 1% (*v*/*v*) crystal violet was added and allowed to stand at room temperature for 5 min. The stained wells were rinsed thoroughly with water to remove unbound crystal dye, and the remaining bound stain was solubilized in 100 μL of 33% (*v*/*v*) glacial acetic acid, after which the OD value was measured at 600 nm. Three biological replicates were performed for each strain, and the experiments were repeated three times. Strains with OD__test_/OD__control_ ≥ 2 were classified as strong biofilm formers.

The efflux pump phenotype was evaluated by comparing the MICs of cefepime against β-lactam-resistant *E. coli* strains before and after treatment with CCCP (10 μg/mL). The MICs were determined as described above. The experiments were repeated three times. Strains exhibiting ≥4-fold decrease in the MIC to cefepime in the presence of CCCP were classified as having an efflux pump-positive phenotype [27].

The β-lactamase production was determined by enzyme-linked immunosorbent assay (ELISA) using a commercial kit following the supplier’s instructions (Jiangsu Meimian Industrial Co., Ltd., Yancheng, China). The assay was performed with three biological replicates per strain, and the entire experiment was conducted three independent times.

The presence of ARGs, including *bla_TEM_*, *bla_CTX-M_*, *acrA, acrB*, and *tolC*, was determined by polymerase chain reaction (PCR). Genomic DNA was extracted using a boiling method. Briefly, a 2 mL of bacterial sample (approximately 1.0 × 10^9^ CFU/mL) was centrifuged at 12,000 rpm for 5 min at 4 °C. The supernatant was discarded, and the pellet was resuspended in 200 μL of 1× TAE buffer. The cell suspension was boiled for 10 min, and then immediately chilled on ice for 10 min. After another centrifugation step under the same conditions, the supernatant was collected and used as the DNA template for PCR. Primers (Table S1) were designed based on sequence from published literature [28,29,30] and synthesized by Sangon Biotech Co., Ltd. (Shanghai, China). The 20 μL PCR mixture contained 10 μL of 2× Hieff^®^ Ultra-Rapid HotStart PCR Master Mix (with Dye) (Yeasen Biotechnology Co., Ltd., Fuzhou, China), 1 μL of each forward and reverse primer, 2 μL of DNA template, and 6 μL of ddH_2_O. Amplification was performed under the following conditions: initial denaturation at 94 °C for 3 min; 30 cycles of denaturation at 94 °C for 10 s, annealing at 49–55.7 °C (gene-specific) for 20 s, and extension at 72 °C for 10 s; and a final extension at 72 °C for 5 min.

The colonization ability of these β-lactam-resistant strains was evaluated using a murine intestinal colonization model. Twelve specific pathogen-free (SPF) C57BL/6 mice were randomly assigned to four groups (A–D), with three mice in each group. The mice were maintained under the aforementioned conditions and subjected to a 7-day acclimation period. On day 8, streptomycin was administered to the mice in drinking water (1 g/L). On day 9, the water was replaced with antibiotic-free drinking water. Subsequently, Mice in groups A and B were inoculated with one β-lactam-resistant strain (strain C26), while groups C and D received the other resistant strain (strain C29). Inoculation was performed via oral gavage once daily for three consecutive days; each dose consisted of 2.0 × 10^7^ CFU of bacteria in a volume of 0.2 mL bacterial suspension (1.0 × 10^8^ CFU/mL). Thereafter, mice in groups B and D were administered MT (20 mg/kg) via oral gavage, while mice in groups A and C received an equal volume of sterile normal saline. Both treatments were given twice daily for 10 days. On day 10 after the last administration, fecal samples were collected from each mouse and processed individually. Briefly, each sample was resuspended in 1 mL of sterile PBS (pH 7.2). The suspension (20 μL) was plated on MacConkey agar containing 0.5 × MIC of cefepime. After overnight incubation at 37 °C, presumptive colonies were analyzed by enterobacterial repetitive intergenic consensus (ERIC)-PCR to compare their genetic profiles with those of the β-lactam-resistant strains (strains C26 and C29). Genomic DNA was extracted using the boiling method as previously described. Primers (Table S1) were designed based on sequence from published literature [31] and were synthesized by Sangon Biotech Co., Ltd. (Shanghai, China). The PCR mixture (25 μL) contained 12.5 μL of 2 × Hieff^®^ Ultra-Rapid HotStart PCR Master Mix (with Dye), 1 μL of each primer, 2 μL of DNA template, and 8.5 μL of ddH_2_O. Amplification was performed as follows: initial denaturation at 94 °C for 5 min; 35 cycles of denaturation at 94 °C for 30 s, annealing at 55 °C for 3 min, and extension at 72 °C for 2 min; and a final extension at 72 °C for 7 min.

### 4.3. Determination of MT-Induced Changes in MIC

For the 24 *E. coli* strains resistant to all seven tested β-lactam antibiotics, the MICs of these β-lactams were determined after treatment with MT at a concentration of 4096 μg/mL in vitro. Briefly, 100 μL of exponentially growing bacterial culture was incubated with an equal volume of double-strength LB broth containing 8192 μg/mL of MT at 37 °C for 48 h. Aliquots (2 μL) of each culture were transferred to 2 mL of fresh LB broth and grown until the exponential growth phase (OD_600_ = 0.5). MICs were determined using the method described above for the screening of β-lactam-resistant *E. coli* strains. The experiments were repeated three times independently.

For the two *E. coli* strains meeting all six previously defined screening criteria (strains C26 and C29), the MICs of cefepime and cefquinome were determined after treatment with various concentrations of MT in vitro. Briefly, 100 μL of exponentially growing bacterial culture was incubated with an equal volume of double-strength LB broth containing two-fold serial dilutions of MT (0, 1024, 2048, 4096, 8192 μg/mL; 0 μg/mL as control) at 37 °C for 48 h. Aliquots (2 μL) of each culture were sampled at 6, 12, 24, and 48 h, transferred to 2 mL of fresh LB broth, and grown until the exponential growth phase (OD_600_ = 0.5). MICs were determined using the method described above for the screening of β-lactam-resistant *E. coli* strains. The experiments were repeated three times independently.

Subsequently, in the murine intestinal colonization model, strains C26 and C29 were treated with MT. The MICs of cefepime and cefquinome against these strains were then determined ex vivo. Thirty SPF C57BL/6 mice were randomly assigned to ten groups (A–J), with three mice per group. Intestinal colonization with strains C26 and C29 was performed according to the procedures for screening β-lactam-resistant *E. coli* strains. Mice in groups A to E were inoculated with strain C26, while those in groups F to J were inoculated with strain C29. Thereafter, mice in groups B and G, C and H, D and I, and E and J received MT (20 mg/kg) by oral gavage twice daily for 2, 4, 8, and 10 days, respectively. Control groups (A and F) received an equal volume of sterile normal saline twice daily for 10 days. Fecal samples were collected from each mouse within 24 h after the last administration. Strains C26 and C29 were isolated and identified via ERIC-PCR as previously described. The two strains were grown in 2 mL of fresh LB broth until the exponential growth phase (OD_600_ = 0.5). MICs were determined using the method described above for the screening of β-lactam-resistant *E. coli* strains.

### 4.4. Determination of MT-Induced Changes in Bacterial Morphology and Growth

The morphology of strain C26 was examined after in vitro treatment with various concentrations of MT. Briefly, an exponentially growing bacterial culture was incubated with MT (0, 512, and 2048 μg/mL; 0 μg/mL as control) at 37 °C for 24 h. Then, the bacterial morphology was observed using scanning electron microscopy (SEM, Hitachi High-Technologies Co., Tokyo, Japan).

The growth curves of strains C26 and C29 were monitored after in vitro treatment with MT. Briefly, 100 μL of exponentially growing bacterial culture was incubated 100 mL of LB broth containing two-fold serial dilutions of MT (0–8192 μg/mL) at 37 °C for 24 h. Aliquots (200 μL) of the culture were sampled at 0, 1, 2, 4, 6, 8, 10, 12, and 24 h, and OD was measured at 600 nm. The experiments were repeated three times independently.

### 4.5. Determination of MT-Induced Change in Biofilm Formation Ability

The biofilm formation ability of strains C26 and C29 was evaluated after in vitro treatment with MT. Briefly, an exponentially growing bacterial culture was incubated with various concentrations of MT (0–4096 μg/mL; 0 μg/mL as control) at 37 °C for 24 h. Thereafter, aliquots (2 μL) of each culture were transferred to 2 mL of fresh LB broth and grown until the exponential growth phase (OD_600_ = 0.5). Biofilm formation was quantified as described previously for the screening of β-lactam-resistant *E. coli* strains. Three independent experiments were performed, each with three biological replicates per strain.

For the in vivo evaluation, the procedures for developing the murine intestinal colonization model, exposing strains C26 and C29 to MT, and for subsequent isolation, identification, and cultivation of the bacteria were consistent with those used for the screening of β-lactam-resistant *E. coli* strains and the determination of MT-induced changes in MIC. Biofilm formation was quantified as described previously. Three independent in vivo experiments were conducted, each with three biological replicates (mice) per group.

### 4.6. Determination of MT-Induced Change in β-Lactamase Production

The β-lactamase production of strains C26 and C29 was determined after in vitro treatment with MT. Briefly, an exponentially growing bacterial culture was incubated with various concentrations of MT (0–4096 μg/mL; 0 μg/mL as control) at 37 °C for 24 h. Aliquots (2 μL) of each culture were transferred to 2 mL of fresh LB broth and grown until the exponential growth phase (OD_600_ = 0.5). The β-lactamase production was quantified by ELISA as described for the screening of β-lactam-resistant *E. coli* strains. Three biological replicates were set for each strain, and the assays were repeated three times.

For the in vivo evaluation, the procedures for developing the murine intestinal colonization model, exposing strains C26 and C29 to MT, and for subsequent isolation, identification, and cultivation of the bacteria, were consistent with those used for the screening of β-lactam-resistant *E. coli* strains and the determination of MT-induced changes in MIC. β-lactamase production was quantified as describe previously. Three independent in vivo experiments were conducted, each with three biological replicates (mice) per group.

### 4.7. Transcriptome Sequencing

Transcriptome sequencing of the β-lactam-resistant *E. coli* strain C26 after in vitro treatment with MT was performed by Huarong Kehui Biotechnology Co., Ltd. (Fuzhou, China). A total of six samples were subjected to transcriptome sequencing, including three treated with MT at 2048 μg/mL and three untreated controls. Briefly, an exponentially growing bacterial culture was incubated with various concentrations of MT (0 and 2048 μg/mL; 0 μg/mL as control) at 37 °C for 24 h. Thereafter, total RNA was extracted using TRIzol reagent (Invitrogen, Carlsbad, CA, USA) according to the manufacturer’s instructions, followed by quantification using a Qubit^®^2.0 Fluorometer (Life Technologies, Carlsbad, CA, USA). Strand-specific cDNA libraries were constructed using the Illumina TruSeq Stranded Kit (Illumina, San Diego, CA, USA), with subsequent sequencing performed on an Illumina HiSeq sequencing platform (Illumina, San Diego, CA, USA). The raw reads were processed by removing adapter sequences and low-quality reads using Fastp v0.23.4. The clean reads were mapped to the reference genome of *E. coli* using Bowtie (v2.2.5, Johns Hopkins University, Baltimore, MD, USA). Gene expression levels were quantified using RSEM (v1.2.12, University of Wisconsin-Madison, Madison, WI, USA). The DEGs were estimated using the DEGseq method [32]. An adjusted *p*-value (Benjamini–Hochberg FDR) ≤ 0.05 and absolute fold change > 2 were used as thresholds for identifying DEGs. Functional enrichment analysis was performed using Cluster and JavaTreeView based on GO and KEGG (http://www.geneontology.org/; http://www.genome.jp/kegg/, accessed on 11 April 2026) databases.

To validate the transcriptomic data, qRT-PCR was used to determine the expression levels of three biofilm-related DEGs (*ycgR*, *pgaB*, and *pgaD*) in strain C26 after in vitro treatment with MT. Additionally, the expression levels of two β-lactamase-encoding genes (*bla_TEM_* and *bla_CTX-M_*), which were not identified as DEGs, were also examined by qRT-PCR in the same MT-treated samples. Total RNA was extracted from MT-treated strains using an Eastep^®^ Super Total RNA Extraction kit (Promega (Beijing) Co., Ltd., Beijing, China) according to the manufacture’s instruction. Then, cDNA was synthesized using NovoScript^®^Plus All-in-one 1st Strand cDNA Synthesis SuperMix (gDNA Purge) (Novoprotein Scientific Inc., Suzhou, China) following the manufacturer’s protocol. qRT-PCR was performed with Eastep^®^ qPCR Master Mix (Promega Biotechnology Co., Ltd., Beijing, China) on a Gentier 96R Real-Time PCR System (Xi’an Tianlong Science & Technology co., Xi’an, China). The qRT-PCR primers (Table S2) were designed based on sequence from published literature [33,34,35] and synthesized by Sangon Biotech Co., Ltd. (Shanghai, China). The qRT-PCR system included 10 μL of 2 × NovoStart^®^Universal Fast SYBR qPCR SuperMix, 0.5 μL of forward primer, 0.5 μL of reverse primer, 1 μL of cDNA template, and 8 μL of RNase free water. The qRT-PCR protocol consisted of an initial 30-s denaturation step at 95 °C, followed by 40 amplification cycles each comprising two steps: 5 s of denaturation at 95 °C and 30 s of combined annealing/extension at 60 °C. The program concluded with a standard melting curve analysis to verify amplicon specificity. This involved incubation at 95 °C for 15 s and 60 °C for 1 min, followed by a continuous temperature increase from 60 °C to 95 °C with continuous fluorescence acquisition. Representative amplification curves for each target gene are provided in Supplementary Material (Figure S1). The *dxs* gene was used as an internal control, and relative quantification was performed by the 2^−ΔΔCt^ method [36]. Each sample had three technical replicates measured per target gene in every independent experiment.

### 4.8. Data Analysis

All results were presented as mean ± standard deviation (SD) unless otherwise specified. Data were analyzed using GraphPad Prism software (v8.0.1, GraphPad Software, Inc., San Diego, CA, USA). Statistical significance among multiple groups was determined by one-way or two-way Analysis of Variance (ANOVA) followed by Dunnett’s test. Differences between two groups were evaluated using Student’s *t*-test. Statistical significance was set at *, *p* < 0.05; **, *p* < 0.01; *** *p* < 0.001.

## 5. Conclusions

In summary, we demonstrated that MT effectively restores the susceptibility of β-lactam-resistant *E. coli* to cefepime and cefquinome in both in vitro and in vivo settings. The resistance-reversing effect is associated with the suppression of biofilm formation and β-lactamase production. Transcriptomic and qRT-PCR analyses further linked these phenotypic changes to the downregulation of key biofilm-related genes (*ycgR*, *pgaB*, *pgaD*) and β-lactamase-encoding genes (*bla_TEM_* and *bla_CTX-M_*). While these findings highlight the therapeutic potential of MT as a resistance-reversing adjuvant, further studies are needed to evaluate its effect on other resistance mechanisms, such as efflux pumps and PBPs, and to validate its efficacy using a broader panel of clinical isolates.

## Figures and Tables

**Table 1:** Susceptibility of 24 β-lactam-resistant *E. coli* strains to seven β-lactam antibiotics determined by broth microdilution method.

Strains No.	MIC (μg/mL)						
	Amoxicillin	Cefazolin	Cefaclor	Cefotaxime	Ceftiofur	Cefepime	Cefquinome
C3	>2048	2048	1024	1024	2048	64	1024
C4	>2048	2048	1024	1024	512	512	1024
C6	>2048	2048	2048	2048	2048	512	2048
C7	>2048	2048	2048	2048	2048	1024	2048
C9	>2048	1024	1024	1024	1024	1024	1024
C10	>2048	>2048	2048	2048	>2048	2048	2048
C11	>2048	2048	2048	2048	>2048	512	2048
C12	>2048	>2048	2048	2048	1024	2048	2048
C13	2048	2048	2048	2048	>2048	512	2048
C14	>2048	2048	1024	2048	1024	1024	2048
C15	>2048	>2048	2048	2048	>2048	256	2048
C16	>2048	>2048	2048	2048	>2048	512	2048
C19	>2048	2048	2048	2048	>2048	1024	2048
C20	>2048	1024	1024	1024	2048	32	512
C21	>2048	2048	2048	2048	>2048	1024	1024
C22	>2048	2048	2048	2048	>2048	1024	2048
C23	>2048	2048	2048	2048	>2048	256	1024
C24	>2048	2048	2048	2048	>2048	512	2048
C25	>2048	1024	2048	512	>2048	512	1024
C26	>2048	1024	512	512	1024	128	256
C27	>2048	2048	2048	2048	>2048	512	1024
C28	>2048	2048	2048	2048	>2048	512	256
C29	>2048	1024	512	512	1024	256	256
C30	>2048	>2048	2048	2048	>2048	1024	512

**Table 2:** Biofilm-forming ability of 24 β-lactam-resistant *E. coli* strains assessed by crystal violet staining.

Strains No.	Ratio of OD__test_ to OD__control_	Biofilm-Forming Ability
C3	1.48	Weak
C4	1.27	Weak
C6	1.40	Weak
C7	1.33	Weak
C9	1.19	Weak
C10	2.56	Strong
C11	1.29	Weak
C12	3.77	Strong
C13	1.13	Weak
C14	1.11	Weak
C15	1.12	Weak
C16	1.26	Weak
C19	1.11	Weak
C20	6.81	Strong
C21	1.09	Weak
C22	1.21	Weak
C23	1.14	Weak
C24	1.35	Weak
C25	2.00	Strong
C26	4.23	Strong
C27	1.08	Weak
C28	1.16	Weak
C29	3.73	Strong
C30	1.05	Weak

**Table 3:** Efflux pump phenotype of 24 β-lactam-resistant *E. coli* strains evaluated using the CCCP inhibition assay.

Strains No.	Ratio of MIC__Control_ to MIC__CCCP treatment_ ^a^	Efflux Pump Phenotype
C3	4	Positive
C4	2	Negative
C6	1	Negative
C7	1	Negative
C9	1	Negative
C10	1	Negative
C11	1	Negative
C12	1	Negative
C13	2	Negative
C14	4	Positive
C15	0.5	Negative
C16	0.5	Negative
C19	1	Negative
C20	4	Positive
C21	2	Negative
C22	1	Negative
C23	0.5	Negative
C24	0.5	Negative
C25	1	Negative
C26	4	Positive
C27	0.5	Negative
C28	0.5	Negative
C29	8	Positive
C30	1	Negative

^a^: The efflux pump phenotype was determined based on the ratio of MIC__Control_ to MIC__CCCP treatment_. A ≥ 4-fold decrease in MIC in the presence of CCCP (which dissipates the proton motive force required for efflux pump function) was considered indicative of an efflux-positive phenotype.

**Table 4:** β-lactamase production in 24 β-lactam-resistant *E. coli* strains quantified by ELISA.

Strains No.	β-Lactamase Content (pg/mL)
C3	52.61 ± 0.80
C4	65.26 ± 0.80
C6	58.93 ± 0.45
C7	88.20 ± 1.04
C9	92.25 ± 0.90
C10	85.94 ± 0.80
C11	95.52 ± 0.58
C12	88.97 ± 0.49
C13	80.81 ± 0.52
C14	88.63 ± 0.55
C15	81.95 ± 0.73
C16	76.04 ± 0.47
C19	76.45 ± 0.84
C20	69.74 ± 0.64
C21	82.73 ± 0.58
C22	78.86 ± 0.65
C23	84.29 ± 0.77
C24	74.45 ± 1.19
C25	65.20 ± 0.72
C26	71.97 ± 0.52
C27	81.20 ± 0.64
C28	63.27 ± 0.65
C29	74.57 ± 0.63
C30	68.24 ± 0.51

**Table 5:** Carriage of 5 antibiotic resistance genes in 24 β-lactam-resistant *E. coli* strains analyzed by PCR amplification.

Antibiotic Resistance Genes	Strains Harboring the Antibiotic Resistance Genes
*bla_TEM_*	C3, C6, C15, C16, C19, C20, C21, C22, C23, C24, C25, C26, C27, C28, C29, C30
*bla_CTX-M_*	C15, C26, C29
*acrA*	All strains
*acrB*	All strains
*tolC*	All strains

**Table 6:** MIC changes in seven β-lactams against 24 β-lactam-resistant *E. coli* strains after MT treatment measured by broth microdilution assay.

Strains No.	Amoxicillin	Cefazolin	Cefaclor	Cefotaxime	Ceftiofur	Cefepime	Cefquinome
C3	No change	No change	No change	No change	No change	2-fold ↓	No change
C4	No change	2-fold ↓	No change	8-fold ↓	8-fold ↓	32-fold ↓	16-fold ↓
C6	No change	No change	No change	No change	No change	No change	No change
C7	No change	No change	No change	No change	2-fold ↓	16-fold ↓	8-fold ↓
C9	No change	No change	No change	No change	No change	8-fold ↓	4-fold ↓
C10	No change	No change	No change	No change	No change	64-fold ↓	32-fold ↓
C11	No change	No change	No change	No change	No change	8-fold ↓	8-fold ↓
C12	No change	No change	2-fold ↓	2-fold ↓	8-fold ↓	128-fold ↓	32-fold ↓
C13	No change	No change	2-fold ↓	No change	No change	32-fold ↓	32-fold ↓
C14	No change	No change	16-fold ↓	16-fold ↓	16-fold ↓	64-fold ↓	64-fold ↓
C15	No change	No change	No change	No change	No change	8-fold ↓	8-fold ↓
C16	No change	No change	No change	No change	No change	4-fold ↓	16-fold ↓
C19	No change	No change	No change	No change	No change	8-fold ↓	16-fold ↓
C20	No change	No change	No change	8-fold ↓	No change	4-fold ↓	16-fold ↓
C21	No change	No change	No change	No change	No change	8-fold ↓	8-fold ↓
C22	No change	2-fold ↓	2-fold ↓	No change	No change	64-fold ↓	64-fold ↓
C23	No change	No change	2-fold ↓	No change	No change	16-fold ↓	8-fold ↓
C24	No change	No change	No change	No change	No change	32-fold ↓	16-fold ↓
C25	No change	No change	No change	No change	No change	2-fold ↓	2-fold ↓
C26	No change	2-fold ↓	2-fold ↓	2-fold ↓	8-fold ↓	32-fold ↓	32-fold ↓
C27	No change	No change	No change	No change	No change	8-fold ↓	8-fold ↓
C28	No change	No change	No change	No change	No change	8-fold ↓	2-fold ↓
C29	No change	2-fold ↓	2-fold ↓	2-fold ↓	8-fold ↓	32-fold ↓	8-fold ↓
C30	No change	No change	No change	No change	No change	No change	No change

Note: ↓ represents a reduction in MIC value, and “X-fold ↓” indicates that the MIC decreased by X-fold aftere MT treatment.

## Data Availability

All data generated or analysed during this study are included in this published article [and its Supplementary Information Files].

## Supplementary Materials

The following supporting information can be downloaded at: https://www.mdpi.com/article/10.3390/antibiotics15050494/s1, Table S1: PCR primers used in this study; Table S2: qRT-PCR primers used in this study. Figure S1. Representative qRT-PCR amplification curves for *ycgR* (A), *pgaB* (B), *pgaD* (C), *bla_TEM_* (D), *bla_CTX-M_* (E) and *dxs* (F). The blue lines represent the untreated control group, and the green lines represent the MT-treated group.

## Abbreviations

The following abbreviations are used in this manuscript:

ARGs	Antibiotic Resistance Genes
CCCP	Carbonyl cyanide 3-chlorophenylhydrazone;
CLSI	Clinical and Laboratory Standards Institute
DEGs	Differentially Expressed Genes
E. coli	Escherichia coli
ELISA	Enzyme-Linked Immunosorbent Assay
ERIC-PCR	Enterobacterial Repetitive Intergenic Consensus Polymerase Chain Reaction
KEGG	Kyoto Encyclopedia of Genes and Genomes
LB	Luria–Bertani
MH	Mueller-Hinton;
MIC	Minimum Inhibitory Concentration;
MT	Matrine;
PBPs	Penicillin-Binding Proteins
PBS	Phosphate-Buffered Saline
PCR	Polymerase Chain Reaction
PGA	poly-N-acetyl-glucosamine
qRT-PCR	Quantitative Real-Time Polymerase Chain Reaction
SD	Standard Deviation
SPF	Specific Pathogen-Free
TAE	Tris-Acetate-EDTA
