# Supplementary material for: Matrine Restores Porcine-Origin β-Lactam-Resistant Escherichia coli to Cefepime and Cefquinome: Association with Impaired Biofilm Formation and β-Lactamase Production
Source: Antibiotics (Basel). 2026 May 14;15(5):494. doi: 10.3390/antibiotics15050494 (PMC13203826; doi:10.3390/antibiotics15050494)
Supplement: Supplementary file 1 [file antibiotics-15-00494-s001.zip › antibiotics-4284347-supplementary.pdf]

Supplementary

Table S1. PCR primers used in this study

Gene	Primer sequence (5'–3')	Annealing temp (°C)	Produce size (bp)	Source
bla$_{TEM}$	ATGAGTATTCAACATTTCCGTG<br>TTACCAATGCTTAATCAGTGAG	52	861	[30]
bla$_{CTX\text{-}M}$	CGTCACGCTGTTGTTAGGAA<br>ACGGCTTTCTGCCTTAGGTT	55	781	[30]
acrA	TTAAGACTTGGACTGTTCAGGC<br>ATGAACAAAAACAGAGGGTTTACG	52.5	1194	[28]
acrB	CGAACTGGACGACTACACG<br>TGCAACCGACTCAAGCC	55.7	591	[29]
tolC	ATGAAGAAATTGCTCCCCATT<br>TCAGTTACGGAAAGGGTTATGA	49	1482	[28]
ERIC	ATGTAAGCTCCTGGGGATTCAC<br>AAGTAAGTGA CTGGGGTGAGCG	55	-	[31]

Table S2. qRT-PCR primers used in this study

Gene	Primer sequence (5'–3')	Produce size (bp)	Source
bla$_{TEM}$	GGGAACCGGAGCTGAATGAA<br>TTGTTGCCGGGAAGCTAGAG	125	This study
bla$_{CTX\text{-}M}$	CACCAATGATATTGCGGTGA<br>GTTGCGGCTGGGTAAAATAG	77	[33]
ycgR	GGCAGTCAAGCCGAAGACAACATC<br>ACGACAAATCATACAGGCGGAAACG	277	This study
pgaB	AAACATCCCTCAGGCTAAAGAC<br>CATTCAGTTGTAATAGGCTCATCC	128	[34]
pgaD	TCTGCTGACGGGTTATTACTG<br>TTGCGGCGTATATTGGTAGG	163	[34]
dxs	CGAGAAACTGGCGATCCTTA<br>CTTCATCAAGCGGTTTCACA	113	[35]

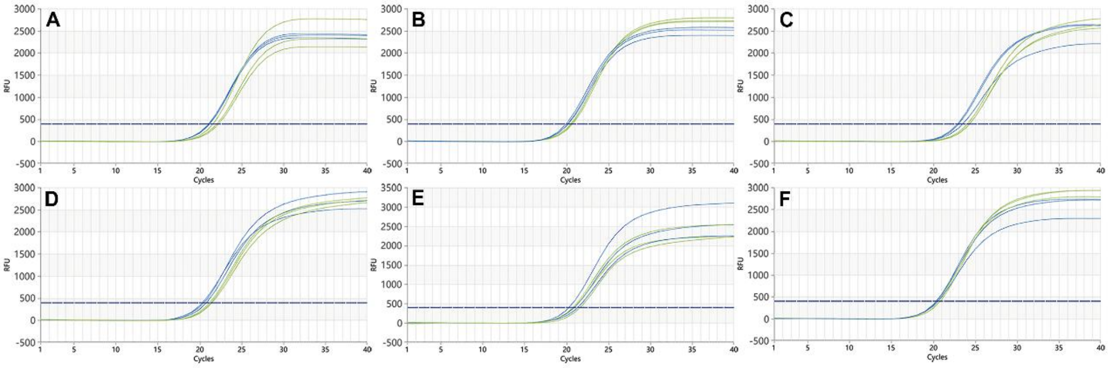

Figure S1. Representative qRT-PCR amplification curves for *ycgR* (A), *pgaB* (B), *pgaD* (C), bla_{TEM} (D), bla_{CTX-M} (E) and *dxs* (F). The blue lines represent the untreated control group, and the green lines represent the MT-treated group.
